# Impacts of diabetes mellitus in the workplace: an integrative review of occupational and economic factors

**DOI:** 10.47626/1679-4435-2025-1509

**Published:** 2026-01-02

**Authors:** Jessica Iracema Diogo Luís, Rogério Muniz de Andrade, João Silvestre Silva-Junior

**Affiliations:** 1 Departamento de Medicina Legal, Bioética, Medicina do Trabalho e Medicina Física e Reabilitação, Faculdade de Medicina, Universidade de São Paulo, São Paulo, SP, Brazil

**Keywords:** diabetes mellitus, occupational health, occupational medicine, risk factors, productivity., diabetes mellitus, saúde do trabalhador, medicina do trabalho, fatores de risco, produtividade.

## Abstract

Diabetes mellitus is a highly prevalent chronic condition worldwide and has a substantial impact on workers’ quality of life and productivity. Its consequences extend beyond clinical manifestations and reach broader social and economic dimensions. Additionally, the workplace can function as a risk factor for the development or worsening of the disease. The aim was to examine the impacts of diabetes mellitus in occupational settings and identify the main work-related factors associated with its occurrence and productivity outcomes. This integrative review was conducted through searches in the Virtual Health Library and PubMed using Portuguese and English descriptors related to diabetes, occupational health, risk factors, and workplace medicine. Studies published in English or Portuguese and relevant grey literature were included. The findings were organized into two thematic categories: economic impact and occupational factors associated with diabetes. Diabetes mellitus was associated with substantial productivity losses, increased absenteeism, presenteeism, and early retirement. Occupational factors such as shift work, long working hours, chronic stress, low job control, and effort-reward imbalance were identified as determinants of type 2 diabetes. A scarcity of studies addressing how workplace conditions influence the management of type 1 diabetes was observed. It was concluded that diabetes mellitus should be recognized as a significant occupational health concern. Implementing workplace health policies, organizational interventions, and health-promotion programs may contribute to disease prevention and to preserving workers’ functional capacity.

## Abstract (pt)

O diabetes mellitus é uma doença crônica de alta prevalência global, que compromete a qualidade de vida e a produtividade dos trabalhadores. Seus impactos extrapolam o campo clínico, alcançando dimensões sociais e econômicas relevantes. Além disso, o ambiente de trabalho pode atuar como fator de risco para o desenvolvimento ou agravamento da doença. O objetivo foi analisar os impactos do diabetes mellitus no contexto ocupacional e identificar os principais fatores laborais associados à sua ocorrência e aos desfechos produtivos. Esta revisão integrativa foi conduzida com buscas nas bases Biblioteca Virtual em Saúde e PubMed, utilizando descritores em português e inglês relacionados a diabetes, saúde ocupacional, fatores de risco e medicina do trabalho. Foram incluídos estudos publicados em inglês ou português, bem como literatura cinzenta pertinente ao tema. Os resultados foram organizados em duas categorias temáticas: impacto econômico e fatores ocupacionais associados ao diabetes. O diabetes mellitus mostrou-se associado a perdas significativas de produtividade, maior absenteísmo, presenteísmo e aposentadoria precoce. Fatores ocupacionais como trabalho em turnos, jornadas prolongadas, estresse crônico, baixa autonomia e desequilíbrio esforço-recompensa foram identificados como determinantes do diabetes tipo 2. Observou-se escassez de estudos sobre a influência do ambiente laboral no manejo do diabetes tipo 1. Concluiu-se que o diabetes deve ser reconhecido como um agravo ocupacional relevante. A implementação de políticas de saúde no trabalho, intervenções organizacionais e programas de promoção da saúde pode contribuir para a prevenção da doença e para a preservação da capacidade laborativa dos trabalhadores.

## INTRODUCTION

Diabetes mellitus (DM) is a complex, chronic disease requiring continuous medical care and comprehensive risk-reduction strategies that go beyond glycemic control. Health education and ongoing support for self-management are essential to empower individuals living with DM, prevent acute complications, and reduce the risk of long-term adverse outcomes.^1^

Although several risk factors for DM are well described and, in many cases, modifiable, the management of the disease in occupational settings still lacks clear definitions.^2^ Work-related factors may act as additional determinants for the development of type 2 diabetes mellitus (T2DM). In a study of workers from a public hospital in Belo Horizonte, Lúcio et al.^3^ found that long working hours, night shifts, multiple jobs, lack of regular meal and rest breaks, and chronic exposure to stress were associated with increased risk of T2DM, although few studies have specifically examined this relationship.

Currently, DM affects approximately 422 million adults worldwide, and this figure is projected to reach about 592 million by 2035. Because of current epidemiological trends, it is unlikely that the United Nations target of halting the rise in diabetes prevalence among adults by 2025 will be achieved.^4^

The growing prevalence of DM among younger individuals indicates that the disease will become increasingly common in people of working age. Consequently, employment and work productivity among individuals with DM are central issues not only for patients and their families but also for employers and policymakers.^5^

The global burden of DM is substantial, with marked effects on labor-market participation, productivity, and economic costs. In addition to increasing premature mortality, DM compromises quality of life and work performance. Evidence shows that diabetes imposes a considerable economic burden in countries with different income levels. In India, for example, DM accounted for 8.5 million excess deaths, 42.7 million years of life lost, and 89.0 million productivity-adjusted life years (PALY), resulting in an estimated loss of 2.6 trillion US dollars in gross domestic product (GDP).^6^ Similarly, in China, DM led to 4.1 million excess deaths, 22.7 million years of life lost, and 75.8 million PALY, with an economic impact also estimated at 2.6 trillion US dollars.^7^

In the United States, in 2007, the costs associated with DM were estimated at 174 billion US dollars: 116 billion in direct costs (personal expenditures, medications, and health services) and 58 billion in indirect costs related to productivity loss. This loss can be expressed through absenteeism (time away from work due to illness), presenteeism (reduced productivity while at work), early retirement (before the legal retirement age), and combinations of these forms.^8^

People living with DM are also at higher risk of unemployment and early exit from the labor market, which may result in reduced income, financial hardship, and negative effects on self-esteem. For employers, diabetes represents a major challenge because of productivity losses related to absenteeism, presenteeism, and premature retirement.^8^

In 2023, the Brazilian Ministry of Health issued Ordinance No. 1,999, which updated the national list of work-related diseases and formally recognized DM as a work-related condition. This measure acknowledges that DM can be caused or aggravated by occupational risk factors, particularly among workers exposed to irregular shifts or high levels of stress, and reinforces the central role of work in shaping the health of the working population.^9^

Since the relevance and timeliness of this topic, the present study aims to describe the impact of DM on work and to identify its occupational risk factors.

## METHODS

This bibliographic review was conducted between July and November 2024 using the Virtual Health Library and PubMed databases. The following descriptors were used in English: “diabetes mellitus,” “occupational medicine,” “risk factors,” and “occupational health”; and in Portuguese: “diabetes mellitus,” “medicina do trabalho,” “fatores de risco,” and “saúde ocupacional.”

We included studies that addressed the relationship between DM and the work environment and were published in Portuguese or English. We excluded duplicate articles, studies whose objectives were not aligned with the purpose of this review, abstracts without full-text availability, and publications in languages other than English or Portuguese. Extracted data were organized into two main thematic categories: economic impact and occupational factors associated with DM.

**Figure 1:**
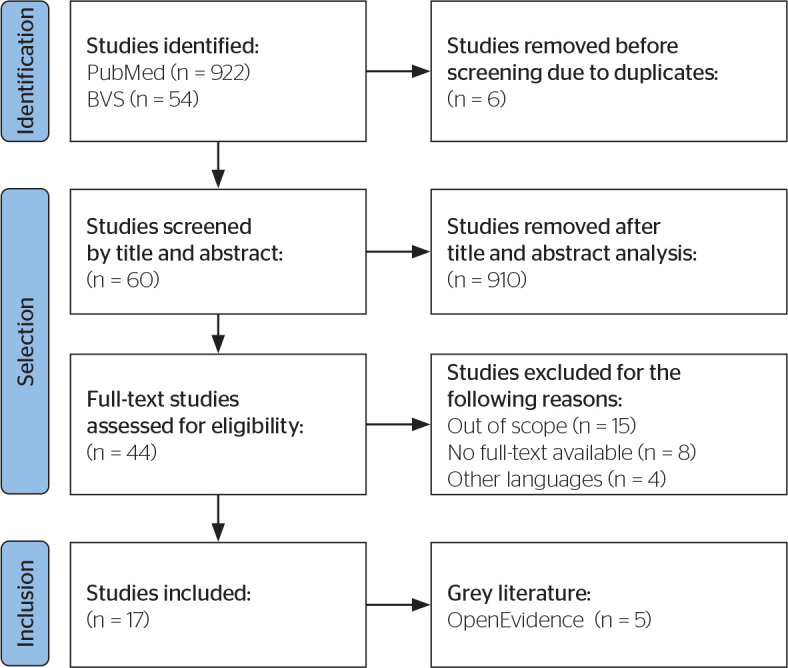
Article selection process according to the Preferred Reporting Items for Systematic Reviews and Meta-Analyses checklist. VHL = Virtual Health Library.

In addition to the articles indexed in the selected databases, grey literature was also incorporated as a complementary source. Five documents that were not subjected to traditional peer review but offered relevant contributions on the impacts of DM in the workplace and on occupational risk factors associated with the disease were analyzed (Figure 1).

## RESULTS

### ECONOMIC IMPACTS

In Brazil, the economic cost of DM in 2016 was estimated at 2.15 billion US dollars, of which 70.6% corresponded to indirect costs related to premature mortality, absenteeism, and early retirement.^10^ In Sub-Saharan Africa, specifically in South Africa, DM resulted in the loss of 13 million PALY, representing an economic impact of 223 billion US dollars in GDP.^11^

A global systematic review on the economic costs of T2DM showed that direct costs are generally higher than indirect costs, although both are substantial. In lowand middle-income countries, a significant portion of this burden falls directly on patients through out-of-pocket treatment expenses.^12^ In Singapore, for example, the total economic cost per working-age adult was estimated at 5,646 US dollars in 2010, with projections reaching 7,791 US dollars by 2050; most of this increase was attributed to losses of productivity.^13^

Because DM predominantly affects individuals of working age, its direct consequences on productivity are a major concern. Losses of productivity arise from absenteeism (absence from work due to illness) and presenteeism (reduced efficiency while at work). The high prevalence of DM in economically active populations, combined with elevated rates of complications and subsequent functional impairment, can lead to substantial productivity losses.^14^

This scenario imposes a significant economic burden on countries, expressed through reduced individual income, lower tax revenue, and declines in GDP, while also increasing pressure on healthcare systems. Although data on the productivity-related impact of DM remain relatively limited, scientific interest in this topic has grown. A recent survey conducted in the United States showed that work performance is significantly impaired in individuals with DM, and that this impairment is directly associated with disease duration and the presence of comorbidities, especially depression.^14^

Approximately 58 billion US dollars are lost annually in the United States due to DM, considering unemployment, reduced productivity, permanent disabilities, and premature deaths. In low-income countries, this impact tends to be proportionally even greater, as premature mortality, including among people of productive age, is more frequent.^14^

### OCCUPATIONAL FACTORS

Multiple occupational factors play an important role in the prevalence of T2DM. A large study conducted in Sweden showed that vehicle drivers and manufacturing workers have significantly higher rates of T2DM compared with university professors and physiotherapists. This difference has been attributed to factors such as high workload, low job control, and physically demanding work environments. These occupational groups also exhibited higher rates of behavioral risk factors, including overweight and smoking.^15^

Working conditions are recognized as potential stressors capable of negatively affecting workers’ health.^16^ Occupational stress is a determinant of several health problems, including cancer, depression, metabolic syndrome, chronic fatigue syndrome, sleep disorders, burnout syndrome, and DM.

High stress levels are associated with changes in glycemia. In stressful situations, the body activates physiological responses that include increases in heart rate and respiratory rate as well as increased blood glucose levels. Although these changes may be reversible when stress is reduced, continuous exposure to chronic stress contributes to the development of DM, particularly in individuals with genetic susceptibility or other metabolic risk factors.^17^

The association between job strain and the development of DM is biologically plausible because stress activates the hypothalamic-pituitary-adrenal axis and leads to cortisol release. Cortisol stimulates hepatic gluconeogenesis and reduces insulin sensitivity in peripheral tissues, promoting hyperglycemia.^18^

One of the most widely used models for evaluating stressful working conditions is the Demand-Control-Social Support model. In this framework, “demand” refers to the psychological workload, whereas “control” refers to workers’ autonomy over the content and volume of their tasks. These dimensions are generally categorized as high or low, producing four combinations: high strain (high demand and low control), active work (high demand and high control), passive work (low demand and low control), and low strain (low demand and high control).^19^ In the Whitehall II study, high-strain work environments were associated with a 60% higher risk of T2DM, and this risk doubled when low social support was also present.^20^

Exposure to psychosocial stressors, such as high demands and low decision latitude, may also heighten the risk of DM, particularly in occupations marked by heavy workloads and low rewards.^21^

The effort-reward imbalance model, proposed by Siegrist,^22^ conceptualizes psychosocial stress at work as a mismatch between the effort invested and the rewards received. Excessive effort includes behaviors such as heightened competitiveness, disproportionate commitment to tasks, and hostile attitudes in the workplace. Inadequate rewards refer to lack of recognition, limited professional advancement, and insufficient career opportunities. Evidence suggests that this imbalance is associated with the development of T2DM, particularly among men.^23^

### SHIFT WORK

#### Direct mechanisms

Workers whose routines hinder the adoption of healthy behaviors, particularly those related to diet and physical activity, may be more vulnerable to developing DM. This is often the case for individuals exposed to long working hours, night shifts, and, in many situations, multiple jobs, without regular meal or rest periods and frequently subjected to high levels of stress and anxiety. Recent studies show that circadian rhythm disruption and occupational stress are associated with an increased risk of developing T2DM, independent of traditional risk factors such as body weight, diet, physical activity, and family history.^16^

Several biological mechanisms may explain the relationship between shift work and DM. First, this work organization pattern interferes with the synchrony of the light-dark cycle and with regular sleep and eating patterns, generating circadian misalignment. Circadian disruption may accelerate the development of T2DM in predisposed individuals. Second, shift work frequently alters sleep schedules, promoting disturbances such as poor sleep quality and subsequent chronobiological disruption. Evidence suggests that insufficient and low-quality sleep contributes to the development and worsening of insulin resistance.^24^

#### Indirect mechanisms

Two recent reviews report growing evidence that overtime work and extended work hours are associated with cardiovascular diseases, poorer self-rated health, and fatigue. Working more than 11 hours per day, compared with a regular workday, has been associated with a nearly threefold higher risk of myocardial infarction and approximately a fourfold higher risk of non-insulin-dependent DM.^25^

DM can also compromise individuals’ ability to remain in the labor market through several pathways. In some cases, complications such as vision loss, amputations, or mobility limitations directly impair functional capacity. Workplace discrimination experienced by individuals with diabetes represents another limiting factor. Moreover, because DM is frequently associated with other health conditions, comorbidities may create additional barriers to employment retention.^26^

## CONCLUSIONS

DM directly affects workers’ productivity and quality of life, representing a major challenge for the global economy. This review showed that the economic burden of the disease is substantial in both high-income and lowand middle-income countries.

The prevalence of T2DM is strongly associated with occupational factors such as work-related stress, long working hours, and job conditions that hinder the adoption of healthy behaviors. Although type 1 diabetes (T1DM) is an autoimmune condition, the work environment also influences its worsening, particularly when there is no organizational support, continuous exposure to stressors, and irregular rest and meal routines. Nevertheless, there is still a scarcity of studies examining the relationship between T1DM and the occupational context, which represents an important gap to be addressed in the scientific literature.

The findings reinforce that DM is not only an individual health problem but also a collective issue that affects the workforce and directly impacts national economies. Economic losses arising from absenteeism and presenteeism highlight the need for effective strategies to prevent and manage the disease, especially in more vulnerable work settings.

The recognition of DM as a work-related disease by the Brazilian Ministry of Health broadens institutional responsibility for this condition and underscores the importance of public policies aimed at workplace health promotion, surveillance of work-related outcomes, and professional rehabilitation.

Implementing occupational health programs that consider psychosocial job demands, the level of control over work tasks, and the adequacy of working conditions can significantly contribute to reducing the incidence of DM and improving workers’ well-being and productivity.

Therefore, mitigating the impact of diabetes in the workplace requires targeted interventions in the work environment, health-promotion actions, and encouragement of healthy lifestyles, aligned with public policies and organizational strategies. Creating healthy and inclusive workplaces is essential to address the challenges posed by DM and to ensure a sustainable, protected, and productive workforce.
